# The Potential Role of Respiratory Motion Management and Image Guidance in the Reduction of Severe Toxicities Following Stereotactic Ablative Radiation Therapy for Patients with Centrally Located Early Stage Non-Small Cell Lung Cancer or Lung Metastases

**DOI:** 10.3389/fonc.2014.00151

**Published:** 2014-06-25

**Authors:** Alexander Chi, Nam Phong Nguyen, Ritsuko Komaki

**Affiliations:** ^1^Department of Radiation Oncology, Mary Babb Randolph Cancer Center of West Virginia University, Morgantown, WV, USA; ^2^International Geriatric Radiotherapy Group, Tucson, AZ, USA; ^3^Department of Radiation Oncology, University of Texas MD Anderson Cancer Center, Houston, TX, USA

**Keywords:** stereotactic ablative radiotherapy, SABR, central location, non-small cell lung cancer, metastases

## Abstract

Image guidance allows delivery of very high doses of radiation over a few fractions, known as stereotactic ablative radiotherapy (SABR). This treatment is associated with excellent outcome for early stage non-small cell lung cancer and metastases to the lungs. In the delivery of SABR, central location constantly poses a challenge due to the difficulty of adequately sparing critical thoracic structures that are immediately adjacent to the tumor if an ablative dose of radiation is to be delivered to the tumor target. As of current, various respiratory motion management and image guidance strategies can be used to ensure accurate tumor target localization prior and/or during daily treatment, which allows for maximal and safe reduction of set up margins. The incorporation of both may lead to the most optimal normal tissue sparing and the most accurate SABR delivery. Here, the clinical outcome, treatment related toxicities, and the pertinent respiratory motion management/image guidance strategies reported in the current literature on SABR for central lung tumors are reviewed.

## Introduction

In the past, thoracic radiotherapy has constantly been limited by toxicity to the normal tissue, such as the lungs and the esophagus, which hinders dose escalation to the gross disease to a desired therapeutic level (1–3). This is mainly due to the utilization of large planning target volume (PTV) margins to compensate uncertainties from respiratory motion and/or in daily patient set up (4, 5). In recent years, advances in imaging technology have enabled us to not only more accurately delineate the gross tumor volume (GTV) and the clinical target volume (CTV), but also given us more information on the location of tumor in relation to critical structures throughout the entire respiratory cycle (1, 2, 4–7). Thus, a PTV margin reduction is possible through accurate delineation of the internal target volume (ITV), which allows for dose escalation to the gross tumor. Tumor localization can be further verified with additional in-room imaging prior to daily treatment to ensure accurate radiation delivery (8). With image-guided radiotherapy (IGRT), ablative doses of radiation can be delivered to treat early stage non-small cell lung (T1–3, N0, M0) or lung metastases, a technique known as stereotactic ablative radiotherapy (SABR) or stereotactic body radiation therapy (SBRT), with excellent clinical outcome consistently observed (9), while local control of over 80% at 3 years have been observed following SABR for oligometastases to the lungs (10, 11).

Despite the rapid clinical adaptation of SABR worldwide, the feasibility of SABR in the treatment of centrally located lung lesions continues to be controversial. The central location is defined as a region that is within 2 cm of the proximal bronchial tree (12). In a phase II prospective study on SABR for T1–2, N0, M0 NSCLC, 60–66 Gy was delivered in three fractions to the tumor target, and the 2-year freedom from severe toxicity was much higher for peripheral lesions when compared to that for central lesions (83 vs. 54%) (13). A total of 12 Grade 3–5 treatment related toxicities were reported at 4 years in this study of 70 patients (14), 5 of which were Grade 5 toxicities. These consisted of pneumonia (three cases), hemoptysis (one case), and respiratory failure (one case). On the contrary, excellent clinical outcome with reasonable toxicity profile has also been reported by others who used dose fractionation regimens with lower fractional dose and increased number of fractions (9). However, treatment related lethal toxicity following SABR for central lung lesions, such as hemoptysis from SABR-related necrosis in the major airway, is still observed when the organ at risk (OAR) was in the high dose volume even when moderate fractionation schedules have been used (15). Therefore, not only lower fractional dose with increased number of fractions is necessary, but geometric accuracy and avoidance of immediately adjacent OARs from being included in the high dose volume are also critical in achieving optimal target volume dose coverage and OAR sparing in the treatment of central lung lesions with SABR (16). These objectives are further complicated by breathing motion, which leads to variation in tumor location relative to adjacent critical organs throughout the entire respiratory cycle. As a result, a high level of image guidance is required to ensure accurate delivery of ablative doses to the tumor target with the smallest treatment margin possible for optimal OAR sparing. In this situation, a sharp dose gradient at the edge of the PTV to spare the immediately adjacent normal organs from receiving an ablative dose of radiation is also strongly desired. In the following sections, the key components of image-guided SABR will be discussed in relation to clinical experience on SABR for central lung lesions. Furthermore, how currently available respiratory motion management and image guidance techniques are used for safe delivery of SABR for central lung lesions, and how to select patients for SABR in this setting will be explored.

## Clinical Experience with SABR for Central Lung Tumors

The clinical experience in delivering SABR for central lung tumors has been reported together with that for peripheral lung tumors in multiple studies (12–14, 17–29). In general, no statistically significant difference in the clinical outcome based on tumor location was observed following SABR for early stage NSCLC (14, 18, 21, 24, 25, 27–30). As shown previously, the biologically effective dose (BED) appears to be a direct predictor of local control following SABR with increased failures observed when a lower BED is delivered to the tumor target irrespective of tumor location (12, 23, 26). In recent years, a number of studies have reported the clinical experience with SABR for central lung lesions alone (30–36). As shown in Table 1, the local control and overall survival following SABR for centrally located early stage NSCLC appear to be very similar to what has been observed following SABR for early stage NSCLC in general (12). Again, BED appears to be a significant predictor of local control favoring a BED of ≥100 Gy_10_ [Gy calculated using an α/β = 10 Gy, BED = total dose × (1 + fractional dose/(α/β))] to the tumor target (37). These findings corroborate with what have been observed in the studies including both peripheral and central lung tumors as mentioned above. Also suggested by studies listed in Table 1, poorer clinical outcome may be observed in advanced stage/recurrent NSCLC or metastases to the lungs when compared with that for early stage NSCLC.

**Table 1:** **Clinical outcome following SABR for centrally located lung tumors alone**.

Reference	No. of patients	Median age	Histology	Median FU (months)	Dose	Local control	DFS/PFS/CSS	OS	Severe toxicities
Haasbeek et al. (30)	63	74 (47–87)	NSCLC: T1–3, N0, M0	35	60 Gy/8 frx	5 years: 92.6%	5 years-DFS: 71%	5 years: 49.5%	Acute: 1 Grade 3 chest wall pain
									Late: 2 Grade 3 dyspnea
									1 Grade 3 chest wall pain
									1 Grade 3 rib fracture
									2/9 Deaths potentially related to SABR
Nuyttens et al. (31)	56	73 (34–88)	NSCLC: 69.6%; metastases: 30.4%	23	45–60 Gy/5 frx 48 Gy/6 frx	2 years: 76% (early stage NSCLC: 85%)	3 years-CSS (early stage NSCLC): 80%	2 years: 60% (early stage NSCLC: 53%)	Acute: 4 Grade 3 pneumonitis Late: 6 Grade 3 pneumonitis
Rowe et al. (32)	47	72 (41–90)	NSCLC: 59%; metastases: 41%	11.3	50 Gy/4 frx (57%)	Two local failures observed			4 Grade 3 dyspnea within 2–4 months after SABR
									One SABR-related death
Oshiro et al. (33)	21	71 (35–89)	Recurrent/metastatic NSCLC: 95% Stage IA: 1 Stage IV: 8 Recurrent rI: 4 rIIA: 1	19.8	25–35 Gy/1 frx 40–48 Gy/4 frx 40–50 Gy/5 frx 48 Gy/8 frx 50–60 Gy/10 frx 39 Gy/3 frx	2 years: 59.6%	2 years-PFS: 23.8%	2 years-OS: 62.2%	Acute: none Late: 1 Grade 3 productive cough due to bronchial stenosis requiring dilatation 1 year after treatment 1 Grade 3 dyspnea 18 months after SABR, which was preceded by three courses of RT to bilateral tumors One SABR-related death
Unger et al. (34)	20	~(23–82)	Hilar lesions abutting or invading the mainstem bronchus. Metastases: 85%	10	30–40 Gy/5 frx	1 year: 63%		1 year: 54%	Acute: 1 Grade 3 radiation pneumonitis 8 months after SABR One SABR-related death
Milano et al. (35)	53	67 (37–88)	NSCLC: 66.04% Stage I 7 Stage II–III 10 Stage IV (oligomet) 15 Stage rIII: 2 Metastases: 33.96%	10	20 Gy/4 frx 12.5–20 Gy/5 frx 40 Gy/8 frx 30–50 Gy/10 frx 49.5–55 Gy/11 frx 42–48 Gy/12 frx 52 Gy/13 frx 45–54 Gy/18 frx	2 years: 73%		2 years: 44% NSCLC Stage I 72% Stage II–III: 12% Oligomet: 50%	Acute: none Late: 1 Grade 3 pneumonia 10 months after SABR Four SABR-related deaths
Chang et al. (36)	27	~	NSCLC Stage I 48.15% Recurrent 51.85%	17	40–50 Gy/4 frx	Three failures following 40 Gy delivered; no failures in the 50 Gy group			One brachial plexopathy with partial arm paralysis after significant volume of the brachial plexus received 40 Gy

*DFS, disease-free survival; PFS, progression-free survival; CSS, cause-specific survival; OS, overall survival; SABR, stereotactic ablative radiotherapy; oligomet, oligometastasis; frx, fraction*.

Severe toxicities and deaths following SABR for centrally located lung lesions have been reported in many studies, which brought great concern regarding the feasibility of SABR for centrally located lung tumors (13, 14, 17–21, 30, 32–35). In these studies, large fractional dose, and/or failure to exclude OARs immediately adjacent to the tumor target from the high dose volume were frequently observed. Both often associated with deaths due to pulmonary injury or bleeding in areas of necrosis in the immediately adjacent organs, such as the esophagus, or the major airways (Tables 2 and 3). As shown in the Indiana phase II study, which included both peripherally and centrally located NSCLC (T1–2, N0, M0), 8 patients with Grade 3–4 toxicities, and 6 SABR-related deaths were identified among 70 patients after a median follow up of 17.5 months when 60–66 Gy was delivered in three fractions (13). The toxicities were mostly cardio-pulmonary in nature. The rate of severe toxicity (Grade 3–5, CTCAE version 2.0.) significantly correlated with tumor location initially with an 11-fold increase in the risk of severe toxicity associated with central location (13). It suggests that centrally located lesions need to be treated differently even when this correlation lost statistical significance after a median follow up of 50.2 months due mostly to the small number of patients included. Death following treatment of central lung lesions with much lower dose per fraction was initially reported by Onimaru et al. (17). This occurred when the esophagus was not excluded from the high dose volume. It ultimately resulted in death due to hemoptysis as a result of an unhealing esophageal ulcer 5 months after SABR. A hot spot above the prescribed dose on the esophagus was later discovered, which may have contributed to esophageal ulceration.

**Table 2:** **Deaths following SABR for central lung tumors in studies including both peripheral and central lesions**.

Reference	Median FU (months)	No of central lesions/study	Lesions associated with death	Dose schedule associated with death	Cause of death/time of death
Fakiris et al. (14)	50.2	22	A pericarinal and a pericardial NSCLC	60–66 Gy/3 frx	Hemoptysis (19.5 months after SABR) and pericardial effusion
Onimaru et al. (17)	18	9	A 3.5-cm metastasis from melanoma posterior to the R mainstem bronchus	48 Gy/8 frx	Bleeding from an unhealing esophageal ulcer 5 months after SABR
			Esophageal dose parameters		
			Maximum dose: 50.5 Gy		
			Mean dose: 10.6 Gy		
			1 cc dose: 42.5 Gy		
Song et al. (18)	26.5	9	Endobronchial NSCLC in the mainstem bronchus	48 Gy/4 frx	Hemoptysis, aspiration, and pneumonia from treatment induced complete bronchial stricture 13 months after SABR
Stauder et al. (19)	15.8	47	A recurrent NSCLC that is obstructing the L mainstem bronchus (pneumonectomy on the contralateral side 17 years ago)	48 Gy/4 frx	Pulmonary failure caused by progressive bronchial obstruction due to tumor necrosis 7.5 months after SABR
Peulen et al. (20)	12	11	Bilateral hilar metastases from RCC, then R hilar recurrence 3 years later L hilar NSCLC encasing a lobar bronchus Carinal recurrence from esophageal cancer	40 Gy/4 frx, then 40 Gy/5 frx 40 Gy/4 frx to the primary disease followed by 33 Gy/3 frx 13 months later 40 Gy/5 frx following chemotherapy followed by 40 Gy/5 frx 29 months later	Hemoptysis 10 months after second course of SABR Hemoptysis/hemorrhage 6 weeks after second course of SABR A fistula between G-tube and trachea developed 10 months after second course of SABR; local progression 13 months after second SABR was treated with 40 Gy/5 frx, then again 42 Gy/7 frx 8 months later; The patient was found to have developed SVC syndrome due to severe RT induced fibrosis 7 months after third course of SABR and died of an MI during stent placement
Bral et al. (21)	16	17	Peri-bronchial early stage NSCLC	60 Gy/4 frx	Hemoptysis related to Grade 3 dyspnea due to bronchial stenosis. The patient died during stenting

*Frx, fractions; RCC, renal cell carcinoma*.

**Table 3:** **Deaths reported in studies on SABR for central lung tumors only**.

Reference	Dose prescribed	Immediately adjacent organs	Dose to critical organs	Cause of death/time of death
Haasbeek et al. (30)	60 Gy/8 frx	Pericardium overlapping the target volume R hilum	Unknown	Cardiac event 2.5 years after SABR Respiratory failure
Rowe et al. (32)	50 Gy/4 frx to a metastasis from melanoma	L mainstem bronchus	Airway point dose: 54.2 Gy Airway_5cc_ dose: 12.7 Gy (overall: 14.7 Gy)	Hemorrhage with bronchial necrosis in the region of the maximum point dose 10.5 months after SABR
Oshiro et al. (33)	25 Gy/1 frx^a^	Hilum of unknown side	Unknown	Hemoptysis 18 months after SABR
Unger et al. (34)	30–40 Gy/5 frx to an endobronchial lesion from mesothelioma	Unknown mainstem bronchus	Maximum point dose: 49 Gy	Bronchial fistula related, 7 months after SABR
Milano et al. (35)	49.5 Gy/11 frx to one central NSCLC followed by 48 Gy/4 frx 15 months later	Bronchus	Bronchus received 98 Gy cumulatively	Hemoptysis 6.5 months after second course of SABR
	50 Gy/10 frx to one central and one peripheral NSCLC followed by 50 Gy/10 frx to three new central lesions and one bulky recurrence of the previously treated peripheral lesion 11 months later	Bronchus and trachea	Unknown	Dyspnea 2 weeks after second course of SABR
	35–50 Gy/10 frx to five central NSCLC	Bronchus and trachea	Unknown	Bronchitis 6 months after second course of SABR
	35 Gy/14 frx then 18 Gy/6 frx to three central NSCLC and 50 Gy/10 frx to one peripheral NSCLC	Bronchus (0.5 cm from tumor) and trachea (1 cm from tumor)	Unknown	Dyspnea 4 months after SABR

*^a^After previous intra-tracheobronchial brachytherapy to bilateral hilar lesions and SABR to the apical area of the same lobe*.

Treatment related toxicities causing death have also been observed in other studies (18–21). As shown in Table 2, death due to bleeding/hemoptysis has been frequently observed following primary or repeat treatments of central lung lesions with SABR. Bronchial strictures and tissue necrosis have also been frequently encountered following SABR for lesions that were adjacent to or within the airways (18, 19). In one study, partial or complete bronchial strictures have been observed in 8/9 patients with centrally located stage I NSCLC after doses from 40–48 Gy/4 to 60 Gy/3 fractions were delivered (18). In their study, severe pulmonary toxicities associated with partial bronchial stricture were observed after 40 Gy/4 fractions were delivered. In a different study, death due to hemoptysis related to bronchial stenosis was observed after a peri-bronchial lesion was treated with 60 Gy/4 fractions (21). These findings demonstrate the risk for severe toxicity due to SABR-related bronchial stricture, which should be avoided whenever possible.

In studies that evaluated SABR for central lesions only (30–36), the incidence of severe toxicities was low among the patients reported. This may be related to lower fractional dose in the dose fractionation schedules used, patient selection, availability of cutting-edge technology for image guidance, and respiratory motion control, as well as many other factors. Among these studies, 9 deaths were reported following SABR in a total of 287 patients (Table 3). Again, bleeding due to tissue necrosis of the immediately adjacent OARs appears to be a common cause of death. Five deaths occurred after multiple courses of radiotherapy to single or multiple peri-bronchial lesions (33, 35), while one death occurred after SABR was delivered to an endobronchial lesion (34). One potential treatment related death due to a cardiac cause occurred in a patient with underlying cardiac conditions for whom the PTV and the heart overlapped (30). One death due to bronchial necrosis related hemorrhage occurred 10.5 months after SABR to a 5.7-cm metastasis abutting the left mainstem bronchus (32). The area of bronchial necrosis was retrospectively found to have received a maximum dose above the dose prescribed.

Stereotactic ablative radiotherapy for central lung tumors has been shown to be feasible without any treatment related severe toxicities by many as well (22–29). No fractional dose of over 12.5 Gy was used among them, which further supports the need to lower the fractional dose when treating centrally located lesions to avoid severe late toxicities (Table 4). However, SABR may not be the best treatment option for endobronchial lesions as it was frequently found to result in bronchial necrosis related complications, causing death (18, 19, 34). In addition, re-irradiating central lung lesions with hypofractionated dose fractionation regimens needs to be considered very carefully given the already increased risk of normal tissue injury from prior treatment (20, 33, 35).

**Table 4:** **Studies on lung SABR reporting no severe toxicity associated with central location**.

Reference	Median FU (months)	No of central lesions/total lesions	Dose fractionation schedule used	Severe toxicities
Xia et al. (22)	27	9/43	50 Gy/10 frx	None
Guckenberger et al. (23)	14	22/159	48 Gy/8 frx^a^	None
			26 Gy/1 frx	
			37.5 Gy/3 frx	
Baba et al. (24)	26^b^	29/124	44–52 Gy/4 frx	None
Olsen et al. (25)^c^	11	19/130	45–50 Gy/5 frx^a^	None
	16		54 Gy/3 frx	
	13			
Andratschke et al. (26)	21	24/92	35 Gy/5 frx^a^	None
			40 Gy/4 frx^a^	
			30–45 Gy/3 frx	
Takeda et al. (27)	OLTs from CRC 29	33/232	50 Gy/5 frx	None
	OLTs from other origins 15			
	NSCLC 24			
Stephans et al. (28)	15.3	7/94	50 Gy/5 frx^a^	None
			60 Gy/3 frx	
Janssen et al. (29)	13.8	29/65	40–48 Gy/8 frx^a^	None
			37.5 Gy/3 frx	

*^a^Dose fractionation schedule for central lesions*.

*^b^For living patients only*.

*^c^Median FU based on dose fractionation schedule used*.

*OLTs, oligometastases; frx, fractions*.

As suggested by the clinical experience summarized above, the following are of pertinent importance in minimizing the risk of severe toxicities following SABR for central lung tumors: the use of dose fractionation schedules with relatively lower fractional dose while increasing the number of fractions accordingly to maintain an adequate BED; carefully respecting the dose constraints for the immediately adjacent OARs during treatment planning; and validation of accurate tumor localization through daily image guidance to ensure that the immediately adjacent structures are kept outside of the high dose region in the context of respiratory motion. Furthermore, sharp dose gradient at the PTV’s edge through intensity modulation is strongly desired to optimize conformal avoidance of the immediately adjacent OARs when treating central lesions with SABR (12). This makes image guidance even more critical in the delivery of daily treatments. In the following sections, the current available respiratory motion management/image guidance techniques that can be used to optimize the safe and accurate delivery of SABR to treat central lung lesions in the context of the clinical studies described above will be further described and assessed.

## Respiratory Motion Management in Lung SABR

Patient immobilization, respiratory motion management, and appropriate image guidance are closely integrated in thoracic IGRT. Multiple image guidance techniques are currently in use to ensure accurate tumor localization during lung SABR and these are closely related to the strategy for respiratory motion management that is used in conjunction with them. Tumor motion due to respiration in various locations of the lungs has been previously described by Seppenwoolde et al. (4). The greatest motion was observed in lower lobe tumors that were not attached to rigid structures in the cranio-caudal direction (12 ± 2 mm), while the lateral motion appears to be much less (2 ± 1 mm). The tumors were found to be more stable and spending more time in the expiratory phase of respiration. In addition, hysteresis of 1–5 mm has been observed commonly (4).

A more detailed description of respiratory motion can be found in a report by AAPM task group 76 (38), which further illustrates that patients’ breathing patterns are irregular, and are highly variable in magnitude, and period. They not only vary intra- and inter-fractionally, but also vary between different patients. As shown by Wulf et al., a uniform ITV margin of 5 mm in transverse and 10 mm cranio-caudally still led to partial misses of tumor targets in 12–16% of the patients even in the setting of stereotactic body frame usage (39). Therefore, individually accounting for respiratory motion with patients breathing in a repeatable fashion is essential for the most accurate and precise capturing of internal organ motion. Furthermore, tumor location needs to be verified under daily image guidance to ensure appropriate dose distribution during actual treatment to justify small PTV margins for the most optimal OAR sparing.

Respiratory motion management strategies currently in use are usually separated into five different categories: motion encompassing, respiratory gating, breath hold, forced shallow breathing with abdominal compression and breath-synchronized, or real time tumor tracking techniques (38). Among them, motion encompassing techniques to estimate the range of tumor motion have been most commonly used in the treatment of central lesions with SABR (Table 5). These include slow CT scanning, ITV generation with inhalation and exhalation breath hold CTs combined with free-breathing CT, and 4D or respiration corrected CT. A slow CT is generated with a speed that would allow multiple respiratory cycles to be captured per slice to generate a tumor encompassing volume, which depicts tumor location throughout the entire respiratory cycle. This approach is limited by the lack of contrast between tumor from normal tissue when it is located in the vicinity of the mediastinum, the diaphragm, or the chest wall as a result of respiration related blurring. Alternatively, FDG PET registered to the planning CT has been used by some to aid target volume delineation due to the enhanced resolution of tumor in areas of soft tissue associated with image registration; and the volume encompassing effect associated with the relatively slower speed of a PET scan (Table 5). The inhalation and exhalation breath hold CTs have been used to estimate the extremes of breathing motion. Respiration monitoring may be used in this setting to confirm that the breathing range is constant and the ITV generated adequately encompasses the tumor at the time of actual treatment. Both methods provide less detail on tumor motion than 4D CT. As shown in Table 5, 4D CT was used for motion management in 7/14 studies in which motion encompassing techniques were used (19, 23, 25, 29, 30, 32, 36). It can estimate the mean tumor position and the range of tumor motion in relation to adjacent normal thoracic organs with increased sophistication when compared to the other two approaches, which is critical for target volume delineation in central locations of the thorax. The use of 4D CT in the treatment planning of lung SABR has been described in detail by Slotman et al. (40). As shown by Wang et al., 4D CT based target volume delineation consistently resulted in smaller PTV volume in lung SABR, which may potentially lead to an increase in normal tissue sparing (41).

**Table 5:** **Treatment planning, immobilization, and image guidance in SABR for central lung tumors**.

Reference	Respiratory motion management	ITV	FDG PET for target definition	Dose calculation/TPS	Technique	Immobilization	Image guidance
Haasbeek et al. (30)	^e^	Y		−/BrainLab	3D	–	ExacTrac system
Nuyttens et al. (31)	RTT			−/CyberKnife	IMRT	–	Fiducial marker tracking per CyberKnife system
Rowe et al. (32)	^e^	Y		AAA/−	3D, IMRT	Full length vacuum cushion	CBCT
Oshiro et al. (33)	^c^			−/Eclipse (Varian)	3D	Individualized body casts	Gated KV-radiographs
Unger et al. (34)	RTT			Non-isocentric inverse planning algorithm with heterogeneity correction/CyberKnife	IMRT	–	IR emitting external markers and internal fiducial markers used for real time tumor tracking with CyberKnife
Milano et al. (35)	^d^	Y	Y	−/BrainLab	Arcs	–	ExacTrac system
Chang et al. (36)	^e^	Y		−/−	3D	–	CT-on-rail with orthogonal radiographs to confirm isocenter
Fakiris et al. (14)	^a^	Y		−/−	3D	SBF with abdominal compression	Daily treatment guided by external markers on SBF
Onimaru et al. (17)	^b^	Y		3D RTP with heterogeneity correction	3D	No immobilization cradles	Orthogonal radiographs on the first day
Song et al. (18)	,c,d^a,c,d^	Y		−/Render 3D system (Elekta) or Eclipse (Varian)	–	Vacuum fitted SBF	CBCT
Stauder et al. (19)	^e^	Y	Y	−/Eclipse (Varian)	3D	BodyFix vacuum system	CBCT
Peulen et al. (20)	^a^	Y		Pencil beam algorithm with heterogeneity correction/−	3D	SBF with abdominal compression	CT prior to each treatment
Bral et al. (21)	,c^b,c^	Y	Y	−/BrainLab	3D	Low density cradle with IR skin markers on the thorax	ExacTrac-like system using both external and internal markers
Xia et al. (22)	^f^	Y	N1 LN delineation	Body gamma knife planning system	MLC based gamma knife	Vacuum bag from head to pelvis	–
Guckenberger et al. (23)	^e^	Y		Collapsed cone algorithm/−	3D	SBF or BodyFix systems	CT, in-room CT, then CBCT since 2005
Baba et al. (24)	^b^	Y		? AAA/eclipse (Varian)	3D	BodyFix system	–
Olsen et al. (25)	^e^	Y		Superposition convolution algorithm with heterogeneity correction/−	3D	SBF system or alpha cradle	CBCT
Andratschke et al. (26)	^f^	Y		Unknown algorithm with heterogeneity correction/−	3D/arcs	Vacuum couch and low pressure foil	CT prior to each treatment, then CBCT since 2008
Takeda et al. (27)	^f^	Y		Superposition algorithm with heterogeneity correction/XiO (CMS)	DCMAT	Corset	–
Stephans et al. (28)	^b^	Y		Unknown with heterogeneity correction/BrainLab	IMRT	BodyFix system	ExacTrac system
Janssen et al. (29)	^e^	Y		−/−	–	SBF with abdominal compression	CBCT

*^a^Forced shallow breathing with abdominal compression*.

*^b^Inhalation and exhalation breath hold CTs used to generate ITV*.

*^c^Respiratory gating*.

^d^Breath hold/active breathing control

*^e^4D CT*.

*^f^Slow CT*.

*SBF, stereotactic body frame; DCMAT, dynamic conformal multiple arc therapy; RTT, real time tracking; Y, yes; ITV refers to that generated with motion encompassing technique or that accounted for when generating the PTV; –, unknown*.

Other respiratory motion management techniques are also used in the treatment of central lung tumors with SABR. The breath hold technique has been used by Song et al. and Milano et al. in the delivery of SABR, while respiratory gating has been used by Song et al. and Oshiro et al. in their patients (18, 33, 35). Forced shallow breathing with abdominal compression has been commonly used to reduce respiratory motion in the pre-4D CT era, when SABR began to become a treatment option for early stage NSCLC (14, 39, 42). Both deep inspiration breath hold (DIBH) and end expiratory breath hold (EEBH) can be used for the breath hold technique while the DIBH approach can potentially improve the sparing of the normal lung tissue (35, 38). However, breath holding requires a high degree of patient cooperation and is often limited to the delivery of 3D-CRT and step-and-shoot IMRT due to the short duration of breath holding of ≤30 s.

Respiratory gating refers to the delivery of radiation within a particular portion of a patient’s respiratory cycle. The respiratory cycle can be monitored through external respiratory signal or internal fiducial markers, while the gating criteria can be set by either displacement (33), or phase based on a certain pre-set displacement distance or phase window, respectively. This technique requires respiration to be continuously monitored using surrogate markers of breathing motion (18, 33, 43). Although it can potentially spare more normal tissue compared to the motion encompassing method, it requires a high degree of quality assurance to validate the accurate representation of tumor motion by the external signal and the internal fiducial markers (38). In addition, treatment time is increased with gating as radiation is only delivered when the target is in the gated window.

Real time tumor tracking is different from the other techniques of respiratory motion management in that the radiation beam moves in synchrony with the tumor as the patient is breathing. The use of this technique is commonly observed with lung SABR delivery by the CyberKnife (CK, Accuray Corp.), a device that attaches a linear accelerator to a robotic arm to allow for beam adaptation to full three-dimensional motion of the tumor under close image guidance (31, 34). This is achieved through the intermittent monitoring of internal fiducial markers or the tumor itself, coupled with the continuous monitoring of external respiratory markers (44). Although the treated volume can potentially be reduced with this highly automated approach, the treatment time is usually long (60–90 min), and the localization of centrally located lung tumors on in-room x-rays may be difficult without the use of internal fiducial markers (44).

## Image Guidance in the Delivery of Lung SABR

Regardless of the motion management strategy used, image guidance during daily treatment is essential in ensuring the accurate localization of the target volume in relation to adjacent normal structures. This allows for smaller PTV margins to be used, especially for centrally located lung tumors, with optimal dose volume coverage and OAR sparing. Image guidance strategies are on-board, peripheral, or integrated on various treatment delivery systems (1). Despite the ability to achieve very sharp dose gradient for normal structure sparing in SABR for central lung lesions, the clinical use of helical tomotherapy (a image-guided IMRT delivery system integrating a six MV linear accelerator with a helical CT) for this purpose has not be extensively reported (12). However, the first two strategies are widely adopted in SABR delivery.

On-board image guidance is conducted when the imaging device is attached to the actual treatment delivery system. The most commonly used on-board imaging device for the delivery of lung SABR is the cone beam CT (CBCT), which is re-constructed from a series of x-ray projections obtained in a single rotation of the source and detector around the patient (45). In the most commonly available CBCT systems, the imaging axis is chosen to be 90° to the treatment beam. CBCT provides 3D information of the tumor in relation to the critical normal structures for online verification of tumor localization prior to the delivery of daily treatment. It can be obtained with either MV or KV imaging. KV imaging is superior to MV imaging in providing better soft tissue resolution with low to moderate imaging doses, which potentially improves patient set up accuracy and alignment of tumor target volume in relation to adjacent critical structures (46). This may be especially helpful in the treatment of central lung lesions with SABR, as a high degree of anatomical information is necessary for the most optimal tumor localization. However, KV CBCT requires regular quality assurance for the alignment of the imaging and treatment beams (46).

Both 2D and 3D imaging are used in peripheral in-room image guidance strategies. The advantage of using imaging devices not directly attached to the treatment delivery system is that respiratory motion may be monitored during the delivery of radiation. However, they need to be carefully calibrated with the treatment beam’s isocenter to minimize additional geometric uncertainties (1). CT-on-rails/in-room CT has been used for online image guidance with the treatment table moved to the imaging position after the patient is set up on the treatment table. Diagnostic quality CT images can be obtained with this approach for the best resolution of soft tissue structures prior to each treatment. However, additional set up errors may be introduced during patient movement between the imaging and treatment positions (46). Both CBCT and CT-on-rails/in-room CT have been used in image-guided SABR for central lung lesions. These strategies are frequently used with the motion encompassing method of respiratory motion management with low incidence of severe toxicities in the setting of primary irradiation, and tumor not directly involving the normal critical structure at risk (18, 19, 22, 23, 25–27, 29, 32, 36). As shown by Grills et al., small PTV margin accounting for systemic and random error may be consistently maintained when CBCT in conjunction with appropriate immobilization were used during SABR delivery for early stage NSCLC (47). In this study, the PTV margin may be reduced to <5 mm with the patient in a stereotactic body frame and to ~5 mm with a regular alpha cradle. Their findings were corroborated in a study by Guckenberger et al., which showed that the PTV margin can be reduced from 12 to <5 mm when KV CBCT is used in addition to a stereotactic body frame (48). In another study, the mean lung dose and the V_20_ (volume of the normal lung receiving 20 Gy) were reduced by 47–77.3%; while the spinal cord dose was reduced by 55.2–58.5% for central lung lesions when CBCT image guidance was used with active breathing control (a breath hold technique) in the delivery of lung SABR as a result of reduction in treatment set up margins enabled by combining image guidance and respiratory motion management (49). In this study, pre-correction set up margins of 14.1 mm in the cranial–caudal direction was able to be reduced to 4.7 mm, while pre-correction set up margins of ~10 mm in the left–right and anterior–posterior directions were reduced to 3.2 and 3.5 mm, respectively. More recently, 4D CBCT has been under investigation to better capture tumor motion at the time of treatment, which may allow for small PTV margins of within 3 mm (50–52). Although fairly accurate with respiratory motion of <5 mm, 3D CBCT was shown to be less accurate in capturing respiratory motion than 4D CBCT as motion artifacts increase with increased tumor motion (53, 54). In addition, accurate localization of the target volume during daily treatment may provide information for adaptive adjustment of the PTV margin and adaptive planning daily. Further exploration in this area is definitely warranted.

Commonly used 2D-imaging based peripheral strategies, such as the Novalis ExacTrac and Synchrony for CK, usually monitor external markers of respiration continuously with periodical verification of tumor location through x-rays of internal tumor markers. With the Novalis ExacTrac system, respiratory motion can be captured by continuous monitoring of infra-red (IR) reflecting markers attached to the patient’s abdomen, while KV x-rays can be matched to digitally re-constructed radiographs for localization verification of internal tumor markers (43, 54). This system can be used for respiratory gating, which may potentially limit the amount of normal tissue irradiated as the gating window can be limited to as small as 2 mm (43). When used to delivery SABR for central lung tumors (21, 28, 30, 35), low incidence of severe toxicities have been observed when re-irradiation was excluded in general (14, 17–21, 30, 32–35). However, Grade 5 toxicities related or potentially related to SABR for lesions in close proximity to the major airway and the heart were reported with this approach of image guidance (21, 30). This suggests that online correction with 3D imaging may be beneficial in certain situations due to the increased amount of 3D geometric detail of critical normal structures in relation to the PTV it provides to avoid non-intended inclusion of critical structures in the high dose volume.

Real time tumor tracking of the CK system is accomplished in a way that is very similar to the ExacTrac system (43, 44). With the Synchrony system, the internal and external marker motions appear to be highly correlated (55). However, external marker based tumor motion prediction are influenced by multiple factors, and its correlation with tumor motion may deteriorate with prolonged treatment duration (56). In addition, a high rate of pneumothorax has been observed after thoracic fiducial marker placement with frequent marker migration (57, 58). Clinically, CK-based SABR has been correlated with excellent clinical outcome (59). It was used to deliver SABR for central lung tumors with only one Grade 5 toxicity encountered when an endobronchial lesion in the mainstem bronchus was treated to the prescribed dose among a total of 76 patients reported in two studies (31, 34). The safe delivery of SABR with CK for central lesions, and especially hilar lesions with relative low incidence of severe toxicity may be due to the fact that relatively smaller PTV can be used with real time tumor tracking as no ITV is needed in this situation (60, 61). When compared with linac-based systems, CK may also be associated with improvement in the sparing of the normal lungs from low dose irradiation for anteriorly located tumors (60, 61). This location-based difference was mostly due to the system’s inability to deliver radiation from underneath the patient. However, these findings suggest that it may provide an advantage in the delivery of SABR for relatively more anterior central lung tumors.

## Conclusion and Future Directions

As shown above, image guidance techniques integrated with respiratory motion management enhances tumor localization in the delivery of SABR for central lung tumors, which are mobile as a result of respiration. As result, very small PTV margin can be safely used to achieve optimal dose coverage of the tumor target and sparing of the adjacent critical normal structures. This makes SABR for central lung lesions feasible when the following criteria are met: primary irradiation of a limited number of lung lesions; dose constraints of the critical structures are strictly respected; and no direct overlap between the PTV and any immediately adjacent OARs. Therefore, the integration of respiratory motion management and image guidance is warranted in future clinical trials on SABR for centrally located lung tumors.

Particle therapy, such as proton therapy, has been increasingly investigated and utilized for the treatment of lung cancer in recent years due to the finite range of charged particles, which may provide an advantage over photon therapy in normal tissue sparing (62). Clinical experience in the delivery of stereotactic body proton therapy has been excellent without any severe toxicity reported in the treatment of central lesions (63, 64). Large smearing margins may be necessary to achieve the most optimal dose distribution in the delivery of passively scattered beams (PT), which may impair OAR sparing in situations of complex geometry (65). Active spot scanning, or intensity modulated proton therapy (IMPT) has been shown to provide a dosimetric advantage in the treatment of central lung lesions over PT and photon therapy (66, 67). However, dose distribution in IMPT is very sensitive to beam and tumor motion, as well as set up uncertainties. Methods to minimize interplay uncertainties have been proposed, which warrants further investigation in the future (65, 68, 69).

## Conflict of Interest Statement

The Guest Associate Editor, Ulf Karlsson, declares that, despite having collaborated with authors Alexander Chi and Nam Nguyen, the review process was handled objectively and no conflict of interest exists. The authors declare that the research was conducted in the absence of any commercial or financial relationships that could be construed as a potential conflict of interest.
